# Toxicity of ubiquitous tire rubber antiozonant *N*-(1,3-dimethylbutyl)-*N*′-phenyl-*p*-phenylenediamine (6PPD) and its transformation product 6PPD-quinone (6PPD-Q) in primary human hepatocytes and liver spheroids

**DOI:** 10.1016/j.bbrep.2025.102199

**Published:** 2025-08-11

**Authors:** Daniel J. Yeisley, Lijun Ren, Katy S. Papineau, Laura K. Schnackenberg, Gonçalo Gamboa da Costa, Tucker A. Patterson, Suzanne C. Fitzpatrick, Qiang Shi

**Affiliations:** a U.S. Food and Drug Administration, National Center for Toxicological Research (NCTR), Jefferson, AR, USA; b U.S. Food and Drug Administration, Human Foods Program, College Park, MD, USA

**Keywords:** 6PPD, 6PPD-Q, Primary human hepatocytes, Liver injury, Spheroids

## Abstract

The tire rubber antioxidant *N*-(1,3-dimethylbutyl)-*N′*-phenyl-*p*-phenylenediamine (6PPD) and its oxidation product 6PPD-quinone (6PPD-Q) were recently found in human bodies. Though 6PPD/6PPD-Q showed species-dependent toxicity in animals, human relevant data are scarce. Here, primary human hepatocytes (PHHs), the gold standard in vitro model for hepatotoxicity, were used for acute and subacute toxicity assessments, with test concentrations normalized to average human serum concentrations (C_ave_). Acute exposure in sandwich cultured PHHs decreased glutathione starting at 100-fold C_ave_ of 6PPD (10 ng/mL) or 500-fold C_ave_ of 6PPD-Q (100 ng/mL), and inhibited albumin starting at 10,000-fold C_ave_ of 6PPD, or 2500-fold C_ave_ of 6PPD-Q. Urea was suppressed by 6PPD-Q, but not 6PPD, starting at 2500-fold C_ave_. Lactate dehydrogenase (LDH) leakage, a measurement of cell death, was unaffected. Subacute exposure of primary human liver spheroids to 6PPD-Q showed no cell death, while 6PPD increased caspase 3/7 activity and LDH leakage and decreased adenosine triphosphate at 50,000-fold C_ave_. Of 10 cytokines involved in hepatotoxicity, interleukin-8 was increased by 6PPD and 6PPD-Q starting from 200- and 50-fold C_ave_, respectively. At 50 to 300-fold C_ave_, the in vivo-relevant concentrations in humans, GSH, caspase 3/7 activity, and interleukin-8 were the only endpoints that were significantly affected by 6PPD and/or 6PPD-Q, and no cell death was observed. These data indicate that 6PPD/6PPD-Q may cause liver dysfunctions and trigger immunotoxicity in heavily exposed individuals but are unlikely to induce significant cell death at regular environmental exposure levels.

## Highlights

• 6PPD/6PPD-Q toxicity was studied in primary human liver cells for the first time.
• 6PPD/6PPD-Q toxicity was tested at concentrations normalized to human serum levels.
• 6PPD/6PPD-Q reduced glutathione, albumin, and urea levels in human liver cells.
• 6PPD/6PPD-Q increased interleukin-8 secretion in human liver cells.
• 6PPD, but not 6PPD-Q, caused significant death in human liver cells.

## Graphical abstract

Some parts of the graphical abstract are adapted from images hosted by Servier MedicalArt and are licensed under a Creative Commons license (CC BY 4.0) https://creativecommons.org/licenses/by/4.0/.

**Figure:**
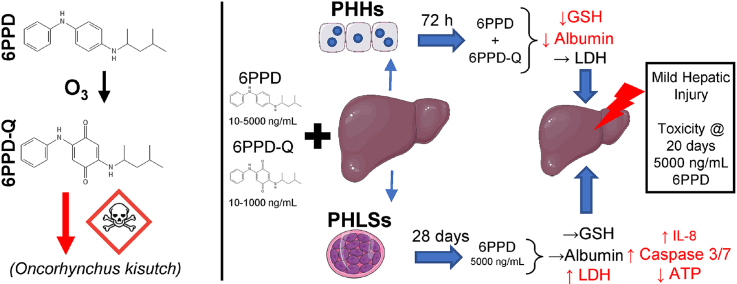

## 1 Introduction

Antiozonants and antioxidants are critical additives to tire rubber that prevent oxidative degradation ensuring prolonged safety during usage, with *p*-phenylenediamines (PPDs), specifically *N*-(1,3-dimethylbutyl)-*N*′-phenyl-*p*-phenylenediamine (6PPD) being the most prevalent by far [1]. However, in 2021 Tian et al. reported that 6PPD-quinone (6PPD-Q), a quinone derived from the environmental transformation of 6PPD, was found to be highly toxic to coho salmon (*Oncorhynchus kisutch)* [2]. The half lethal concentration (LC_50_) of 6PPD-Q was found to be ∼95 ng/L, over 20-fold lower than observed environmental peaks from urban runoff (∼2 μg/L), and is purportedly responsible for the sharp population decline of coho salmon in the Pacific Northwest of the United States [2,3]. While 6PPD-Q was not found to be as acutely toxic to other aquatic organisms in subsequent studies [[4], [5], [6]], the discovery of 6PPD-Q and its toxicity to specific aquatic species sparked major concern regarding 6PPD as an emergent environmental pollutant.

Human exposure to 6PPD and 6PPD-Q comes from many sources with 6PPD and 6PPD-Q having been detected not only in urban runoff, but also in receiving water (peaking post-rainfalls as high as ∼1 μg/L), vehicle dust (156 ng/g), indoor dust (14 ng/g), airborne road dust (>2.9 ng/L) and soil (309 ng/g) [2,[7], [8], [9], [10], [11], [12]]. The main ingress of 6PPD and 6PPD-Q in the environment is through tire wear particles (TWP), a ubiquitous pollutant and one of the primary contributors to global microplastic pollution. TWP-laden dust is highly mobile and persistent within the environment accounting for at least 5−10 % of all oceanic microplastic (MP) and up to >50 % of all MP emissions in some countries [13,14]. 6PPD accounts for 1−2 % by weight of all tires produced over the past 50 years with over 2.2 billion tires produced in 2021 alone; 6PPD-Q makes up ∼5−20 % of the degradation products of 6PPD, indicating potential for significant human exposure due to the prevalence of TWP pollution [15,16]. 6PPD-Q also has a significantly longer hydrolysis half-life than 6PPD which could lead to a greater potential for accumulation once formed [17,18]. Some studies have also begun exploring the bioaccumulation of 6PPD in food products such as fish, honey, and lettuce [19,20].

The scarcity of human data likely stems from the lack of a safety concern over 6PPD due to early rodent data demonstrating lethal toxicity only at exposure levels (oral half lethal dose [LD_50_] ∼1000−3580 mg/kg in mice; dermal LD_50_ > 3000 mg/kg in rabbits) far in excess of those expected during the manufacturing processes (peak values ∼2−6.6 μg/L air) [21,22]. As such, despite 6PPD and 6PPD-containing rubber being widely commercially manufactured since the mid-1960s, little research has shown negative effects in humans beyond a few case studies on dermatitis and potential cross-sensitization in a few individuals [[22], [23], [24], [25]]. Aside from the early manufacturing safety studies in rodents, in vivo animal data on the effects of 6PPD and its newly discovered metabolite 6PPD-Q are relatively sparse but expanding rapidly.

Due to the uncertainty of the impact that 6PPD and 6PPD-Q may have in vivo, recent animal studies have broadly interrogated the compounds, investigating their effects on reproductive organs, the intestinal barrier, the lungs, the brain, the kidneys, the spleen, and the liver [[26], [27], [28], [29], [30], [31], [32]]. Of these, the lung and the liver thus far seem to be the most sensitive organs to these compounds [28,31,32]. In the studies by L. Fang et al. and He et al., 6PPD and 6PPD-Q were found to bioaccumulate in murine liver tissue as well as increase liver weight/volume, triglyceride levels, and levels of alanine aminotransferase (ALT), aspartate aminotransferase (AST), and alkaline phosphatase (ALP) [28,32]. Furthermore, 6PPD-Q was observed to exhibit potential immunogenic effects including shifts in immune-related gene expression and an increase in liver infiltration by recruited macrophages in mice [28,30,32]. However, it is unknown if those animal findings on liver effects are of human relevance – the Guidance documents issued by regulatory agencies from multiple countries suggest that human hepatotoxicity cannot be accurately predicted by standard animal tests (https://www.fda.gov/media/116737/download). In a recently published epidemiology study, Song et al. explored the potential association between 6PPD liver levels and secondary nonalcoholic fatty liver disease wherein a positive association with 6PPD was reported [33]. Together, these studies suggest a potential for 6PPD and 6PPD-Q to cause hepatic injury and warrant further investigation into the effects of these compounds on human livers.

Many in vitro models are available to study human hepatotoxicity, but primary human hepatocytes (PHHs) are generally considered as the gold standard [34]. To examine the possible hepatotoxicity of 6PPD and 6PPD-Q, this study utilized both conventionally cultured pooled (PHHs) and a recently commercialized primary human liver spheroids (PHLSs) model (InSphero), which cocultures PHHs and primary human nonparenchymal cells (NPC). Pooled PHHs in conventional culture can be maintained for 4 days after thawing and were used to evaluate specific hepatocellular effects of 6PPD and 6PPD-Q from acute exposure (3 days of treatment). PHLSs can survive for up to one month without significant loss of function [35] and were thus used to examine the subacute toxicity (28 days of treatment) of 6PPD and 6PPD-Q. The concentrations of 6PPD and 6PPD-Q utilized were based on commonly accepted scaling factors for in vitro to in vivo extrapolation [[36], [37], [38]] and ranged from 50 to 5000-fold of the reported average human serum concentrations (C_ave_) of 6PPD-Q (∼0.2 ng/mL) or 100 to 50,000-fold C_ave_ of 6PPD (∼0.1 ng/mL) [29,33]. Endpoints measured focused on markers of liver injury reflective of both function and cytotoxicity, including lactate dehydrogenase (LDH), albumin, urea, caspase 3/7, reduced glutathione (GSH), cellular adenosine triphosphate (ATP) and a panel of 10 cytokines that are involved in human hepatotoxicity.

## 2 Materials and methods

### 2.1 Chemicals and reagents

6PPD (catalog number: 687875; purity 98.09 %) and 6PPD-Q (catalog number: 687855; purity 98.09 %) were purchased from HPC Standards Inc. (Atlanta, GA, USA). The stock solutions were prepared in dimethyl sulfoxide (DMSO; ATCC, USA, purity >99 %). Acetaminophen (APAP) was purchased from Sigma Aldrich (St. Louis, MO, USA; purity 98.0−102.0 %) and stock solutions were prepared freshly in cell culture medium and sterile filtered.

### 2.2 Cell culture and compound treatment

#### 2.2.1 Conventional sandwich PHH monoculture

The use of primary human liver cells including those for PHH and PHLSs were reviewed and approved by the FDA Institutional Review Board (IRB), which determined that this work does not meet the definition of human subject research and therefore there are no IRB related issues. Cryopreserved CryostaX^TM^ PHHs from a diverse 10-donor pool were acquired from XenoTech (A BioIVT Company, Kansas City, KS, USA; catalog number: HPCH10+; lot number: 2210146) and thawed and plated according to the manufacturer's protocol. All media, medium supplements, and the overlaying matrix (OptiTHAW Hepatocyte Medium, OptiPLATE Hepatocyte Medium, OptiMATRIX, OptiCULTURE, and Penicillin Streptomycin [PS]) were also purchased from XenoTech and handled per the manufacturer's protocol. Briefly, cryopreserved cells were immediately transferred into pre-warmed OptiTHAW medium, centrifuged, and resuspended in OptiPLATE medium before being counted and plated on collagen I coated 24-well plates (Corning; Glendale, AZ, USA) at a density of 1.74 x 10^5^ cells/cm^2^. Cells were allowed to adhere for 4 h then overlayed with OptiMatrix (0.25 mg/mL) prepared in OptiCULTURE Medium and allowed to polymerize overnight. The following day the overlay medium was replaced with OptiCULTURE medium containing test materials or the vehicle control.

#### 2.2.2 InSphero InSight^TM^ primary human liver spheroid microplates

Culture ready 96-well microplates containing 3D Insight^TM^ PHLSs made with hepatocytes from a diverse 10-donor pool (lot number: IPHH_32) and NPCs (Kupffer cells and liver endothelial cells (LECs)) from a single donor (lot number: IPHN_18) were purchased from InSphero (Schlieren, Switzerland) and handled per the manufacturer's protocols. Briefly, upon receiving, the plates were centrifuged, and transportation medium was removed and replaced with proprietary maintenance medium (3D InSight^TM^ Human Liver Maintenance Medium – TOX from InSphero) before resting overnight. Selection and preparation of 6PPD and 6PPD-Q treatments are described in section 3.2.3. On the following day, cell culture medium was removed and PHLSs were treated with 6PPD, 6PPD-Q, APAP (10 mM), a vehicle control (0.1 % DMSO) and a medium only control. APAP treatment was only applied through day 14 due to nearly 100 % cell loss observed on day 14.

#### 2.2.3 PPD and 6PPD-Q treatment and preparation

Stock solutions of 6PPD and 6PPD-Q were generated by dissolving the powdered compounds at a concentration of 10 mg/mL in DMSO and stored at −20 °C. For both PHHs and PHLSs, 6PPD was added at final concentrations of 10, 100, 500, 1000, and 5000 ng/mL and 6PPD-Q at final concentrations of 10, 100, 500, and 1000 ng/mL. These concentrations correspond to 50 to 5000-fold average serum concentration (C_ave_) for 6PPD-Q [29,33]. The test concentration of 6PPD corresponds to 100 to 50,000-fold C_ave_ 6PPD [33]. Exposure to 6PPD and 6PPD-Q were compared to the vehicle control (0.1 % DMSO). For PHLSs, APAP (10 mM) and cell culture medium only (Medium) controls were included.

### 2.3 Endpoint analyses

#### 2.3.1 Extracellular markers – albumin, urea, and LDH

Secreted albumin, urea, and LDH were all measured using commercially available kits. Briefly, albumin was measured via an enzyme-linked immunosorbent assay (ELISA) Human ALB/Serum Albumin ELISA Kit (Sigma-Aldrich) or Human Albumin ELISA Kit (Abcam Inc, Waltham, MA, USA). Urea content in cell culture supernatants was measured using a commercially available colorimetric assay kit, the QuantiChrom^TM^ Urea Assay Kit (BioAssay Systems, Hayward, CA, USA) per the manufacturer's protocol for low urea samples. Limit of detection (LOD) of the assay as reported by the company's protocol is 0.08 mg/dL. As a measure of cell loss/cytotoxicity, LDH activity was measured from cell culture supernatants using a commercially available luminescence kit, the LDH-Glo™ Cytotoxicity Assay (Promega, Madison, WI, USA) per the manufacturer's protocol. Absorbance or luminescence were measured using an Infinite M200 Pro (Tecan Group Ltd., Switzerland) plate reader. Data were normalized to either μg/million cells/day, ng/PHLS/day, or % of the vehicle control.

#### 2.3.2 Intracellular markers – GSH, ATP, and caspase 3/7

GSH, ATP, and caspase 3/7 activity were measured using commercially available luminescent kits, the GSH-Glo™ Glutathione Assay, the CellTiter-Glo® 2.0 Assay, and the Caspase-Glo® 3/7 Assay, respectively, per the manufacturer's protocol (Promega, Madison, WI, USA). For PHLSs, the assays were performed per the manufacturer's protocol scaled to the volume of the spheroid culture plates (70 μL) and samples were read in 384-well plates at a volume of 25 μL. Luminescence was read using an Infinite M200 Pro (Tecan Group Ltd., Switzerland) plate reader. Data were normalized to the vehicle control.

#### 2.3.3 Cytokine levels in culture medium

A panel of 10 cytokines that are involved in immune-driven liver cell damage including interferon gamma (IFN-γ), interleukin one beta (IL-1β), interleukin 2 (IL-2), interleukin 4 (IL-4), interleukin 6 (IL-6), interleukin 8 (IL-8), interleukin 10 (IL-10), interleukin 12 p70 (IL-12p70), interleukin 13 (IL-13), and tumor necrosis factor alpha (TNF-α) was measured using the V-PLEX Plus Proinflammatory Panel 1 Human Kit and the signal was collected using the MESO QuickPlex SQ 120 MM system (Meso Scale Diagnostics LLC, Rockville, Maryland). The manufacturer protocol was followed, except 50 μL of culture medium was used in the assay.

### 2.4 Statistical analysis

Data are reported as the mean ± standard deviation (SD) representing at least three independent experiments (*n* ≥ 3) with *n* being reported in the figure captions. All statistical tests were performed in GraphPad Prism (version 10.1.1). Statistical significance was determined using either a Student's t-test or a one-way analysis of variance (ANOVA) followed by a Dunnett's *post hoc* test (p < 0.05) comparing all treatment groups to the vehicle control. ANOVA followed by a Tukey Honestly Significant Difference (HSD) *post hoc* test was also performed to check select intergroup differences.

## 3 Results

### 3.1 Acute (3-day) toxicity of 6PPD and 6PPD-Q in PHHs in conventional sandwich culture

**Fig. 1:**
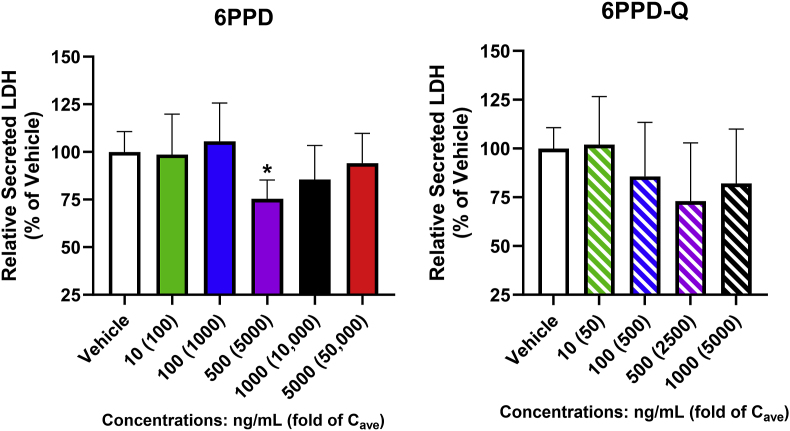
**LDH leakage from PHHs in response to 6PPD or 6PPD-Q.** Sandwich-cultured PHHs were treated with 6PPD and 6PPD-Q for 3 days, and the relative levels of LDH activity in culture medium were measured using a luminescence kit (Promega). ∗p < 0.05 as compared to control. Data are means ± SDs (n = 4–10).

Initial investigation of the potential human hepatotoxicity of 6PPD and 6PPD-Q was performed using PHHs in conventional sandwich culture, in which key liver functions can be maintained for 72 h after compound treatment. Fig. 1 shows that neither 6PPD nor 6PPD-Q caused significant increases of LDH activity in the culture medium as compared to vehicle control (0.1 % DMSO), suggesting that no cell death was induced by 6PPD or 6PPD-Q treatment.

**Fig. 2:**
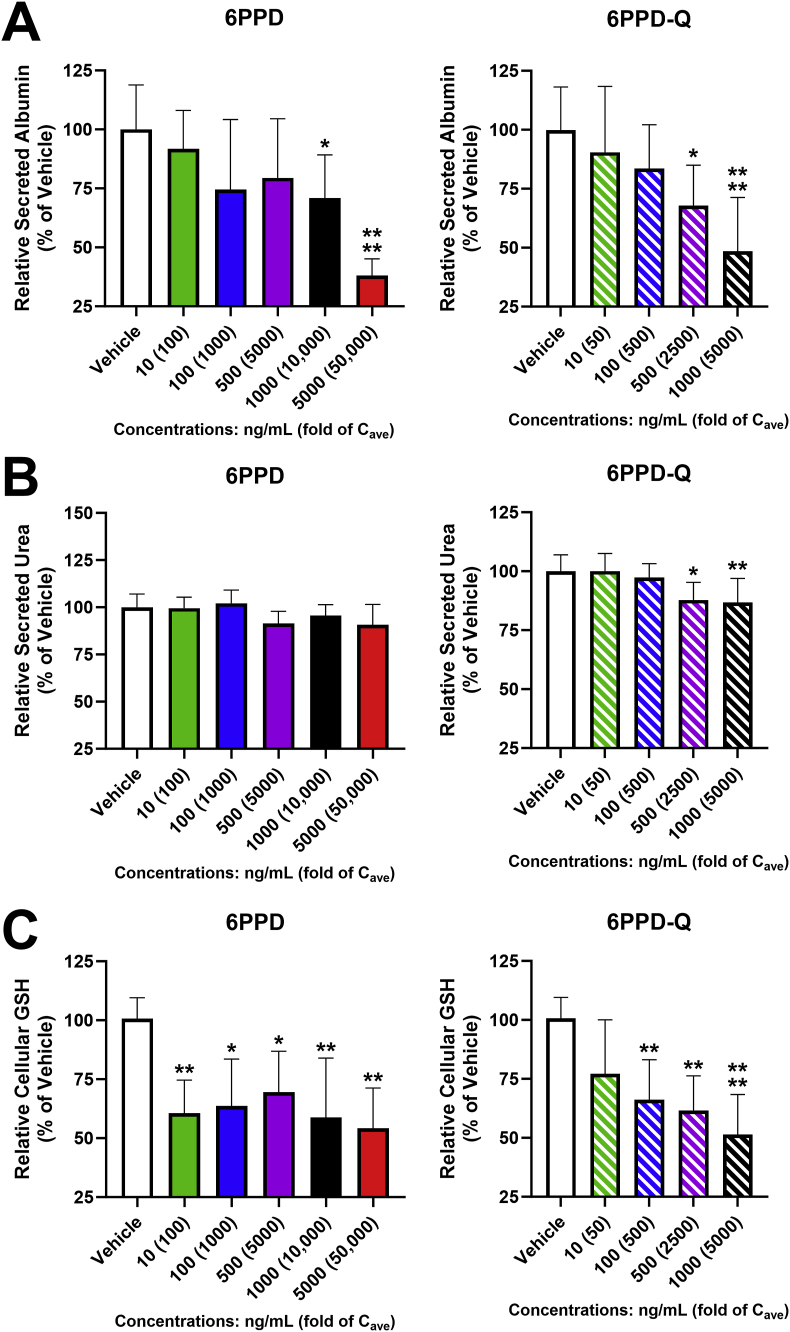
**Functional changes of PHHs in response to 6PPD or 6PPD-Q**. Sandwich-cultured PHHs were treated with 6PPD and 6PPD-Q for 3 days, and cell functions were assessed by (A) albumin levels measured using an ELISA kit (Sigma), (B) urea levels analyzed using a colorimetric assay kit (BioAssay Systems), and (C) GSH levels measured using a luminescent kit (Promega). ∗p < 0.05, ∗∗p < 0.01, ∗∗∗∗p < 0.0001 as compared to control. Data are means ± SDs (n = 3–8).

In terms of endpoints relevant to liver function, both 6PPD and 6PPD-Q significantly decreased albumin production at concentrations of 1000 ng/mL (∼30 %, p < 0.05 [10,000-fold C_ave_ 6PPD]; and ∼52 %, p < 0.0001 [5000-fold C_ave_ 6PPD-Q], respectively) and 6PPD at 5000 ng/mL (∼62 %, p < 0.0001 [50,000-fold C_ave_ 6PPD]) (Fig. 2A) relative to the vehicle control. 6PPD-Q at the lower concentration of 500 ng/mL led to a significant reduction to albumin levels (∼42 %, p < 0.05 [2500-fold C_ave_ 6PPD-Q]). Similarly, 6PPD-Q, but not 6PPD, significantly reduced secreted urea levels in groups exposed to 500 ng/mL and 1000 ng/mL (∼12 %, p < 0.05; and ∼13 %, p < 0.005, respectively) (Fig. 2B). Lastly, both 6PPD and 6PPD-Q significantly reduced GSH concentrations in cell lysates relative to the vehicle control (Fig. 2C). 6PPD reduced levels of GSH at all test concentrations – 10, 100, 500, 1000, and 5000 ng/mL (∼39 %, p < 0.01 [100-fold C_ave_ 6PPD]; ∼37 %; p < 0.05 [1000-fold C_ave_ 6PPD]; ∼31 %, p < 0.05 [5000-fold C_ave_ 6PPD]; ∼42 %, p < 0.01; and 46 %, p < 0.01, respectively). Of note, there were no apparent differences in sensitivity to 6PPD versus 6PPD-Q exposure as at no concentrations were the effects of 6PPD significantly different from the same concentration of 6PPD-Q as evaluated by ANOVA followed by a Tukey HSD *post hoc* test (p < 0.05).

### 3.2 Subacute (28-day) toxicity of 6PPD and 6PPD-Q in PHLSs

**Fig. 3:**
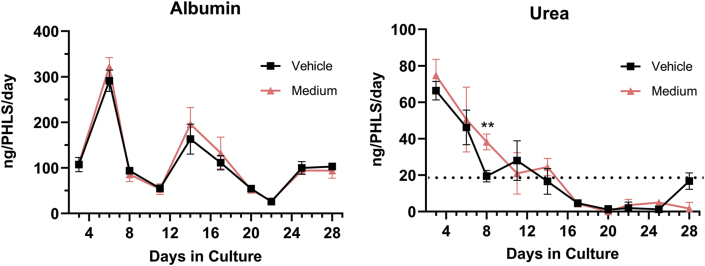
**Time dependent changes of albumin and urea levels in culture medium of PHLSs.** Levels of secreted albumin and urea were measured using cell culture supernatants of untreated PHLSs or those treated with 0.1 % DMSO for 28 days. The dotted line in the urea plot indicates assay limit of detection. ∗∗p < 0.01 as compared to the control. Data are means ± SDs (n = 3–4).

To evaluate the response of multiple liver cell types in coculture following subacute exposure to 6PPD and 6PPD-Q, PHLSs containing PHHs from a diverse 10-donor pool and NPCs (Kupfer cells and LECs) from a single donor were cultured with or without the test compounds for 28 days. A DMSO (0.1 %) vehicle control was compared to blank cell culture medium to ensure the vehicle had minimal impact on the performance of the cell culture system. Levels of albumin and urea in the vehicle control versus the medium control (Medium) were statistically the same on all days except for urea levels on day 8 where the vehicle control was observed to have a transient reduction (∼49 %, p < 0.01) (Fig. 3) relative to the medium only control. These data suggest that DMSO (0.1 %) has negligible impact on PHLS behavior over 28 days relative to spheroids with medium alone.

**Fig. 4:**
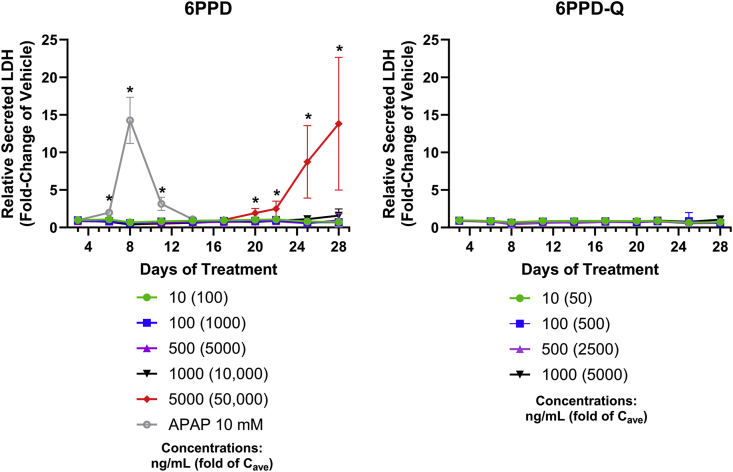
**LDH leakage from PHLSs treated with 6PPD or 6PPD-Q**. PHLSs were treated with 6PPD or 6PPD-Q for 28 days, and LDH activity was measured using culture medium on the specified days. APAP (10 mM) was included as a positive control for LDH activity assay. ∗p < 0.05 as compared to control. Data are means ± SDs (n = 7–24).

Similar to the results in PHH sandwich culture, induction of LDH release was not observed in PHLSs exposed to 6PPD-Q at any time points or test concentrations. However, 6PPD at the highest concentration of 5000 ng/mL (50,000-fold C_ave_ 6PPD) was sufficient to increase LDH release (∼1.91-fold, p < 0.0001) relative to the vehicle control starting on day 20, increasing through day 28 (∼12.80-fold, p < 0.0001) (Fig. 4). The highest observed LDH release values in 5000 ng/mL treated PHLSs is comparable to the maximum release of LDH in those treated with 10 mM APAP (∼13.25-fold, p < 0.0001) relative the vehicle control observed on day 8.

**Fig. 5:**
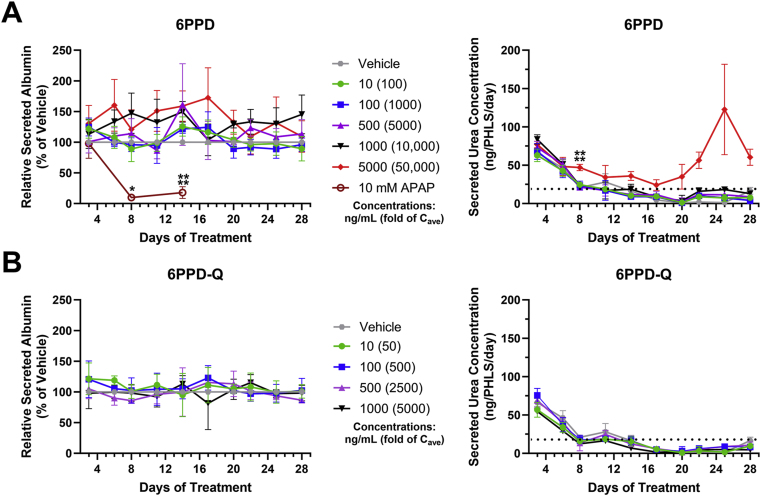
**Functional changes of PHLSs in response to 6PPD or 6PPD-Q.** PHLSs were treated with 6PPD (A) or 6PPD-Q (B) for 28 days, and then levels of albumin and urea were measured using culture medium on the specified days. APAP (10 mM) was included as a positive control for albumin measurement. Dotted lines in the urea graphs indicate the assay limit of detection. ∗p < 0.05, ∗∗∗∗p < 0.0001 as compared to control. Data are means ± SDs (n = 3–8).

In terms of endpoints reflecting basic liver functions, there were no significant decreases in albumin levels in any 6PPP/6PPP-Q groups relative to the control (Fig. 5A). As a positive control, 10 mM APAP decreased albumin levels in cell culture supernatant by up to 90 % (p < 0.0001) as compared to the vehicle control and blank medium. Treatments with 5000 and 1000 ng/mL 6PPD resulted in slight increases in albumin on most days, suggesting the activation of compensatory mechanisms. Urea levels were also increased in PHLSs treated with 5000 ng/mL 6PPD starting on day 8 and peaking on day 25 (Fig. 5A), again reflecting a compensatory reaction. Urea levels in all groups other than 5000 ng/mL 6PPD fell below the LOD of the assay kit on day 14, therefore statistical significance for days 14 through day 28 are not calculable although they were obviously increased in the 5000 ng/mL treated group. 6PPD-Q did not significantly affect levels of albumin and urea relative to the vehicle control over the course of four weeks at any concentration (Fig. 5B).

**Fig. 6:**
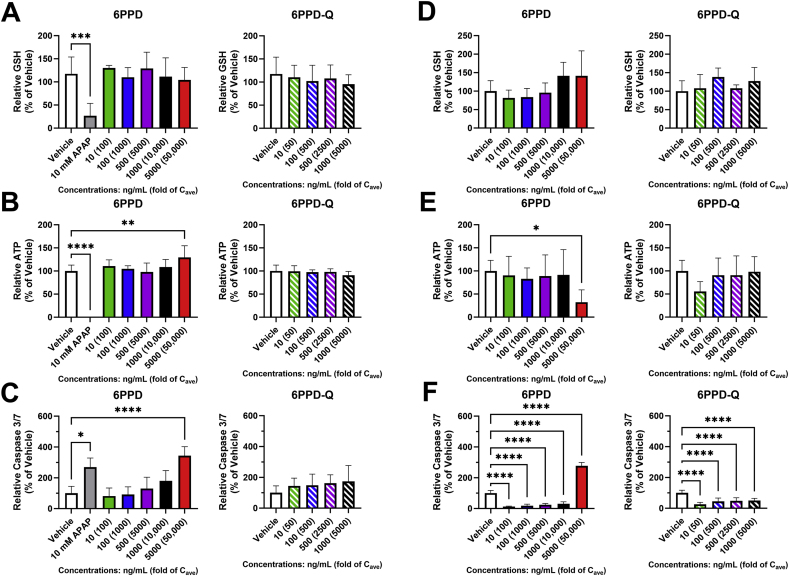
**Effects of 6PPD or 6PPD-Q on cellular GSH, ATP and caspase 3/7 activity in PHLSs.** PHLSs were treated with 6PPD or 6PPD-Q for 28 days, and GSH, ATP and caspase 3/7 activity were measured on day 14 (A–C) and day 28 (D–F). All endpoints were assessed using commercial kits from Promega. APAP (10 mM) was included as a positive control. ∗p < 0.05, ∗∗p < 0.01, ∗∗∗p < 0.001, ∗∗∗∗p < 0.0001 as compared to control. Data are means ± SDs (n = 3–6).

Intracellular endpoints for PHLSs were measured on days 14 and 28 to provide additional mechanistic insight into the impact of 6PPD and 6PPD-Q on liver cells. On day 14, unlike the PHHs in sandwich culture, exposure to 6PPD or 6PPD-Q did not trigger any changes to cellular GSH levels (Fig. 6A). As a positive control, 10 mM APAP alone expectedly reduced cellular GSH levels (∼74 %, p < 0.001) relative to the vehicle control. Similarly, only 10 mM APAP decreased cellular ATP levels (∼99.8 %, p < 0.0001) relative to the vehicle control on day 14, whereas 5000 ng/mL modestly increased ATP levels (∼29 %, p < 0.01) (Fig. 6B), suggesting of a compensatory response was induced by 6PPD in PHLSs. However, on day 14, exposure to 5000 ng/mL 6PPD was observed to increase caspase 3/7 activity (∼243 %, p < 0.0001) (Fig. 6C) relative to the vehicle control. A similar increase was noted in 10 mM APAP treated PHLSs (∼169 %, p < 0.05). 6PPD-Q did not affect ATP or caspase 3/7 activity on day 14 at any concentration. By day 28, there were still no observed changes to GSH levels in 6PPD- or 6PPD-Q- exposed groups (Fig. 6D). However, 5000 ng/mL 6PPD significantly decreased cellular ATP levels (∼68 %, p < 0.05) (Fig. 6E) and increased caspase 3/7 activity (∼120 %, p < 0.0001) (Fig. 6F) relative to the vehicle control. Interestingly, all other treatments of 6PPD and all concentrations of 6PPD-Q reduced caspase 3/7 activity by as much as ∼88 % (p < 0.0001) for 6PPD or ∼75 % (p < 0.0001) for 6PPD-Q, the maximum reduction having been observed in the lowest concentration (10 ng/mL) for both.

**Fig. 7:**
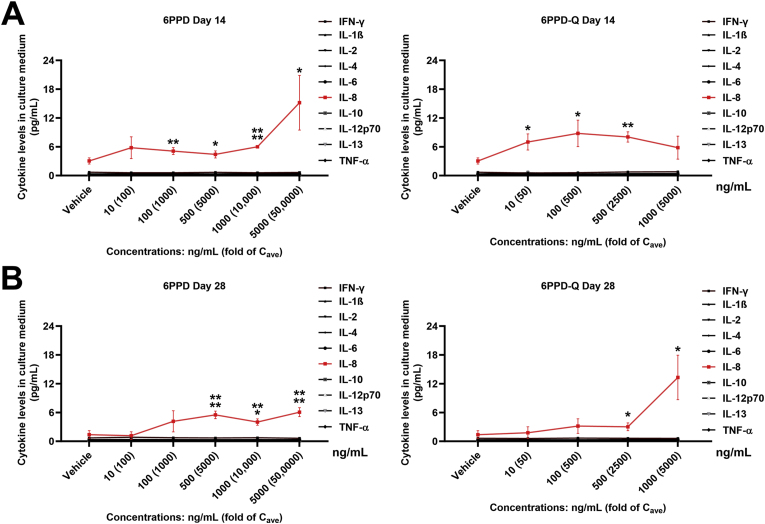
**Effects of 6PPD or 6PPD-Q on 10 cytokines secreted from PHLSs.** PHLSs were treated with 6PPD or 6PPD-Q for 28 days, and 10 secreted cytokines were measured from cell culture supernatants on day 14 (A) and day 28 (B) using a V-PLEX Plus Proinflammatory Panel 1 Human Kit. ∗p < 0.05, ∗∗p < 0.01, ∗∗∗p < 0.001, ∗∗∗∗p < 0.0001 as compared to control. Data are means ± SDs (n = 3).

The effects of 6PPD and 6PPD-Q on a panel of 10 cytokines are shown in Fig. 7. On day 14 of treatment, both 6PPD and 6PPD-Q caused concentration-dependent elevations of IL-8, but not the other 9 cytokines, in the culture medium, with the maximal increase being about 3-fold for 6PPD-Q and 5-fold for 6PPD (Fig. 7A). Significant elevations of IL-8 were even observed at the lowest 6PPD-Q concentrations on day 14 of treatment. These elevations were largely maintained on day 28 of treatment except for those at the lower test concentrations (Fig. 7B), likely because cells were able to adapt to the insult induced by low-concentration treatment. Of note, among the other 9 cytokines, the levels of IL-1β were below the low limit of detection - 0.05 pg/ml according to the kit manufacturer - in all groups. The levels of the remaining cytokines were all readily detectable, but only IL-8 showed significant elevations in response to 6PPD and 6PPD-Q.

## 4 Discussion

Despite the ubiquitous use of 6PPD for decades and the pervasive nature and extent of TWP pollution distribution, there has been limited concern over its impact on the environment or human health. This is likely due to the lack of serious toxicities at peak manufacturing levels, which could be presumed to be the greatest acute human exposure. However, following the discovery that 6PPD-Q — the environmental degradation product of 6PPD — is highly toxic in select salmonids, namely coho salmon, at environmentally relevant concentrations [2], and given the ubiquity of 6PPD and 6PPD-Q through TWP pollution [39], the environmental and human safety of these compounds have become of interest to both the public and regulatory agencies. While the research on 6PPD and 6PPD-Q toxicity is rapidly growing, there are currently gaps surrounding human relevant data. This study seeks to provide some of the earliest human data, based upon PHHs toxicological assessments, which is especially needed for compounds like 6PPD-Q that demonstrate a high degree of species-depended toxicity [6]. The data herein provide novel insights into primary hepatocellular effects of the compounds in both monoculture and mixed liver cell coculture settings during acute – in conventionally cultured PHHs – and subacute exposure – in a 3D spheroid-based method.

Selection of human relevant concentrations in PHH-based models is of critical importance. Though 100-fold of human serum concentrations were widely used in in vitro PHHs models to predict in vivo hepatotoxicity [36], a recent FDA accepted “Letter of Intent” document for the purpose of using a PHH model to predict human hepatotoxicity proposes the use of up to 300-fold serum concentrations (https://force-dsc.my.site.com/ddt/s/ddt-project?ddtprojectid=197). As the selection of in vivo-relevant concentrations in in vitro system is debatable [38], we decided to include the highest testable concentration in cell culture medium. For 6PPD-Q, 1000 ng/mL was determined to be the highest testable concentration, as higher concentrations (5000 and 10,000 ng/mL) were observed to precipitate in medium over time. Similarly, 5000 ng/mL was selected for 6PPD as the highest testable concentrations due to solubility concerns. On the other hand, preliminary studies established that lower concentrations (<10 ng/mL) showed no appreciable effects. Human levels of 6PPD were first reported following a urine analysis performed in 1984 from samples collected from Italian rubber factory workers [40]. Recently, both 6PPD and 6PPD-Q have been found in human urine with detection frequencies ranging from 0 to 82 % and 86–100 %, respectively [[41], [42], [43], [44]]. Notably, pregnant women were observed to have the highest urine levels of 6PPD-Q (∼8.6 ng/mL) [42]. More meaningful for the design of in vitro PHH experiments, human serum concentrations of 6PPD and 6PPD-Q have been very recently reported. Human serum 6PPD had a detection frequency of 14–68 % and range of 0–1.73 ng/mL (mean: ∼0.1 ng/mL) [33,41], and 6PPD-Q had detection frequencies ranging from 3 % to 100 %, with a concentration range of 0–1.1 ng/mL (mean: ∼0.2 ng/mL) [29,33,41]. 6PPD-Q has also been measured in cerebrospinal fluid in a small study with levels averaging 5 ng/mL in 64 % of a healthy cohort (n = 11) [45]. It is worth noting that accurately quantitating 6PPD and 6PPD-Q in serum remains challenging due to lack of high-quality analytical standards and rapid biotransformation, and existing data are limited only in Chinese cities [44,[46], [47], [48]]. To account for uncertainties in quantification, varying exposure levels among population groups, idiosyncratic sensitivities, and limits of solubility, concentrations of 6PPD and 6PPD-Q in the present study were designed to cover a broad range of levels, starting from 50- or 100-fold C_ave_ and extending to 5000- or 50,000-fold C_ave_, respectively.

The highest concentration in PHHs that is considered as highly relevant to in vivo hepatotoxicity is 300-fold serum concentration, and our data show that neither 6PPD nor 6PPD-Q caused apparent cell death at these levels (that is, 30 ng/mL for 6PPD and 60 ng/mL for 6PPD-Q), as determined by cellular ATP, LDH release, and caspase activation (Fig. 1, Fig. 4, Fig. 6C). Therefore, our data suggest that 6PPD or 6PPD-Q is unlikely to cause significant liver cell death at levels currently reported in normal healthy human population. However, cellular GSH level was decreased by 6PPD at 100-fold C_ave_ or 6PPD-Q at 500-fold C_ave_ in PHHs (Fig. 2C), suggesting that 6PPD, but not 6PPD-Q, may lead to functional alterations in vivo in healthy human livers. GSH is among the first line of defense against cellular injury in hepatocytes; a decrease in GSH in the absence of cell death suggests that 6PPD and 6PPD-Q caused mild oxidative stress, but compensatory mechanisms in hepatocytes are activated to cope with the stress and maintain cell survival.

An interesting observation is that IL-8 was significantly elevated by 6PPD-Q at 50-fold C_ave_ or by 6PPD at 200-fold C_ave_ (Fig. 7), concentrations that are highly clinically relevant. IL-8 is produced by many cell types including liver cells and is upregulated by proinflammatory stimuli. One of the main functions of IL-8 is to inhibit apoptosis preserving cell survival [49,50]. This can explain why caspase 3/7 activity, a marker of apoptosis, was decreased by 6PPD and 6PPD-Q in nearly all the tested concentrations (Fig. 6F). At the highest concentrations of 6PPD, IL-8 afforded protection is likely overwhelmed by pro-apoptosis pathways and therefore increased caspase 3/7 activity was observed (Fig. 6F). In humans, increased serum IL-8 levels have been demonstrated in chronic liver diseases such as alcohol-associated hepatitis and metabolic dysfunction-associated steatohepatitis (MASH) [51,52], and recent reports suggested a possible association between 6PPD/6PPD-Q exposure and the development of MASH [32,33,53]. Therefore, it will be worthwhile to investigate the association between IL-8 and 6PPD/6PPD-Q levels in human serum in future investigations. In addition, over production of IL-8 triggers a cascade of immune responses and therefore our data suggest that 6PPD/6PPD-Q may induce immunotoxicity. Future work examining 6PPD/6PPD-Q effects on IL-8 release in non-liver cell types could provide novel insights into the mechanisms of 6PPD/6PPD-Q induced toxicities observed in non-liver organs.

Chemical- or drug-induced human hepatotoxicity is very often idiosyncratic (that is, highly individual dependent) or is sometimes associated with specific population group. Literature reports show that the highest human serum concentrations (C_max_) in some individuals can be 1.73 ng/mL for 6PPD and 1.1 ng/mL for 6PPD-Q [29,33,41]. Our data suggest that cellular GSH or excreted IL-8 was significantly disrupted at 6-fold C_max_ of 6PPD (Fig. 2C) or 9-fold C_max_ of 6PPD-Q (Fig. 7A), respectively. These in vitro findings have the strongest in vivo relevance as the responses were seen at concentrations very close to peak serum levels, and the data imply that individuals with high levels of exposure may develop liver abnormalities related to GSH shortage or IL-8 over production. Patients with pre-existing liver diseases may have decreased capacity to cope with such stress or eliminate xenobiotics and therefore can be more susceptible to 6PPD/6PPD-Q induced toxicities. Indeed, case studies showing that only a few individuals developed contact dermatitis among many exposed to 6PPD-containing rubber can be seen as evidence that 6PPD toxicity can be idiosyncratic [[23], [24], [25]]. In addition, pregnant women were found to have the highest level of 6PPD-Q in urine (∼8.6 ng/mL) [42], though the serum level is unknown. Given the dose-dependent effect of 6PPD-Q, it can be speculated that pregnant women may potentially be at greater risk by developing additional liver dysfunctions such as reductions in albumin and urea production (Fig. 2A and B). Identifying susceptible individuals to 6PPD/6PPD-Q associated liver injury is needed in future studies.

Although a previous study using the same PHLSs technology showed that treatments for 28 days remarkably increased the toxicity of certain test drugs as compared to sandwich cultured PHH [35], subacute exposure to 6PPD or 6PPD-Q in PHLSs over 28 days did not demonstrate greater functional alterations compared to the sandwich cultured PHHs. In PHLSs, albumin and GSH were not decreased at any concentration relative to the vehicle control (0.1 % DMSO) (Fig. 5, Fig. 6), while a 25 %–75 % reduction was observed in sandwich cultured PHH (Fig. 2). However, 6PPD at the highest test concentration indeed triggered significantly increased cell death in PHLSs (Fig. 4) as compared to sandwich cultured PHHs (Fig. 1). A possible explanation for the differential response between sandwich cultured PPHs and PHLSs is that the former has lower compensatory capacity, such as the production of pro-survival cytokines by NPCs, in response to low-concentration treatment. However, with high concentration of 6PPD, PHLSs may become more sensitive to injury due to activation of immune cells [32] or additional duration-dependent injury pathways triggering extensive cell death. Data from PHH sandwich culture and PHLSs provide complementary value in aiding the safety assessment of 6PPD and 6PPD-Q.

This study presents some of the first highly human-relevant experimental data using primary human liver cells towards elucidating the human response to the 6PPD and 6PPD-Q. There have been some other recent in vitro studies utilizing cancer cell lines of human origin, that is, HepG2 and LO2 cells, that investigated the molecular interactions of 6PPD and 6PPD-Q within cells – e.g., effects on amino acid synthesis and interactions with albumin [54,55]. However, the translatability of cancer cell line data to human toxicology is severely limited due to major differences in behavior between HepG2 cells and PHHs [56,57] and LO2 cells being only debatably hepatocyte-like [58,59]. Regardless of the translational values, there are some similarities in responses between PHH- and HepG2 cell-based models, namely the increases in oxidative stress markers [54], which is in line with our observations on GSH alterations, highlighting a direct interaction of 6PPD and 6PPD-Q in oxidative stress pathways. In contrast, Qi et al. and Chen et al. noted that 6PPD and 6PPD-Q were able to induce cell death more readily in cancer cell lines at lower concentrations of 5000- to 20,000-fold C_ave_, as opposed to PHLSs in which we only observed toxicity at 50,000-fold C_ave_ for 6PPD [54,55], suggesting that certain cancer cell lines may be more sensitive than primary human liver cells. A key difference between liver cancer cells lines and PHHs is that the former has extremely low levels of xenobiotic-metabolizing enzymes; therefore, the increased sensitivity of liver cancer cell lines to 6PPD and 6PPD-Q toxicity than PHHs indicates that the hepatic biotransformation of those two compounds is potentially a detoxification process.

As albumin and urea are exclusively synthesized in the liver, our in vitro observations can be validated by examining the correlation between serum levels of 6PPD/6PPD-Q and albumin/urea in human subjects. In addition, investigating if human serum IL-8 is elevated in 6PPD/6PPD-Q exposed individuals can also provide confirmatory evidence for our in vitro findings.

Some limitations of this study are worth noting. While two independent and diverse 10-donor pools of PHHs were utilized in this study, it is possible that specific individuals, including ones belonging to potentially susceptible subpopulations like pregnant women or individuals with pre-existing liver conditions, may have a greater sensitivity to 6PPD or 6PPD-Q than the scope of this study could capture. It will be important to investigate the correlations between serum albumin/cytokine levels and 6PPD/6PPD-Q concentrations among different population groups in future epidemiological studies. In addition, while we observed remarkable increases of caspase 3/7 activities at the highest testable concentration of 6PPD (Fig. 6C and F), complementary assays, such as the terminal deoxynucleotidyl transferase dUTP nick end labeling (TUNEL), need to be developed in PHLSs to confirm the possible apoptosis-inducing effect of 6PPD.

## 5 Conclusions

By using a gold standard in vitro model for hepatotoxicity, this study evaluated the acute (3-day) and subacute (28-day) toxicity of 6PPD and 6PPD-Q at a wide range of concentrations. Our data indicate that these compounds may cause liver dysfunctions (such as a decrease in glutathione, urea, and albumin production) and immunotoxicity (such as over production of IL-8) in normal healthy humans with heavy environmental exposure, but the likelihood of them triggering significant liver cell death is very low. Future investigations to elaborate on the translational value of our in vitro findings are needed to support regulatory decision making to ensure public health is protected from these environmental contaminants.

## CRediT authorship contribution statement

**Daniel J. Yeisley:** Writing – review & editing, Writing – original draft, Methodology, Investigation, Formal analysis, Conceptualization. **Lijun Ren:** Writing – review & editing, Methodology, Investigation. **Katy S. Papineau:** Writing – review & editing, Resources, Methodology, Investigation. **Laura K. Schnackenberg:** Writing – review & editing, Supervision, Resources, Project administration, Investigation, Conceptualization. **Gonçalo Gamboa da Costa:** Writing – review & editing, Supervision, Resources, Funding acquisition, Conceptualization. **Tucker A. Patterson:** Writing – review & editing, Supervision, Resources, Funding acquisition, Conceptualization. **Suzanne C. Fitzpatrick:** Writing – review & editing, Supervision, Resources, Investigation, Funding acquisition, Conceptualization. **Qiang Shi:** Writing – review & editing, Methodology, Investigation, Supervision, Resources, Funding acquisition, Conceptualization.

## Disclaimer

This presentation reflects the views of the authors and does not necessarily reflect those of the U.S. Food and Drug Administration. Any mention of commercial products is for clarification only and is not intended as approval, endorsement, or recommendation. Part of the data were presented as a poster at the 63rd Annual Meeting of Society of Toxicology in Salt Lake City, UT in March 2024 and at the MPS World Summit in Seattle, WA in June 2024.

## Funding sources

Dr. Daniel J. Yeisley is supported in part by both the Research Participation Program, at the National Center of Toxicological Research administrated by the 10.13039/100006229Oak Ridge Institute for Science and Education through an interagency agreement between the U.S. Department of Energy and the U.S. FDA, and the FDA Traineeship (FRST) Program in the Office of the Chief Scientist at the National Center for Toxicological Research, U.S. FDA. This project was funded in part by the Human Foods Program.

## Declaration of competing interest

The authors declare that they have no known competing financial interests or personal relationships that could have appeared to influence the work reported in this paper.

## Data Availability

Data will be made available on request.
